# Genomic Characterization of Dengue Virus in the Ningxia Hui Autonomous Region, China (2019 and 2023)

**DOI:** 10.1093/gbe/evag045

**Published:** 2026-02-28

**Authors:** Longshan Li, Jun Zhan, Dongzhi Yang, Tao Li, Xiaoqiang Sun, Fang Yuan, Min Cao, Wei Zhang, Ting Mu, Jingting Wang, Jianxin Pei, Xueping Ma

**Affiliations:** Department of Virology Laboratory, Ningxia Hui Autonomous Region Center for Disease Control and Prevention, Yinchuan, China; Department of Virology Laboratory, Ningxia Hui Autonomous Region Center for Disease Control and Prevention, Yinchuan, China; Department of Virology Laboratory, Ningxia Hui Autonomous Region Center for Disease Control and Prevention, Yinchuan, China; Department of Virology Laboratory, Ningxia Hui Autonomous Region Center for Disease Control and Prevention, Yinchuan, China; Department of Virology Laboratory, Ningxia Hui Autonomous Region Center for Disease Control and Prevention, Yinchuan, China; Department of Virology Laboratory, Ningxia Hui Autonomous Region Center for Disease Control and Prevention, Yinchuan, China; Department of Virology Laboratory, Ningxia Hui Autonomous Region Center for Disease Control and Prevention, Yinchuan, China; Department of Virology Laboratory, Ningxia Hui Autonomous Region Center for Disease Control and Prevention, Yinchuan, China; Department of Virology Laboratory, Ningxia Hui Autonomous Region Center for Disease Control and Prevention, Yinchuan, China; Department of Virology Laboratory, Ningxia Hui Autonomous Region Center for Disease Control and Prevention, Yinchuan, China; Department of Virology Laboratory, Ningxia Hui Autonomous Region Center for Disease Control and Prevention, Yinchuan, China; Department of Virology Laboratory, Ningxia Hui Autonomous Region Center for Disease Control and Prevention, Yinchuan, China

**Keywords:** DENV-1, phylogenetic analysis, genomic surveillance, human monoclonal antibodies, *C-prM*

## Abstract

Dengue virus (DENV) transmission has risen across China in recent years, but genomic data from inland regions remain scarce. We screened 9 clinical samples collected in Ningxia in 2019 and 2023, obtaining 5 near-complete DENV-1 genomes, and performed phylogenetic reconstruction and molecular clock analyses using a global dataset of 452 DENV-1 sequences to infer spatiotemporal origins; epitope divergence relative to vaccine strains and human monoclonal antibodies was also assessed. The 2019 Ningxia strains clustered within DENV-1 genotype I, clade E, and were closely related to viruses from Hebei Province, whereas the 2023 strain fell within genotype I, clade K, showing the highest similarity to sequences from Yunnan and Guangdong. The most recent common ancestor of the Ningxia strains was estimated to have existed around 1989 (95% highest posterior density: 1985–1993) and is shared with strains from Guangdong, Yunnan, and Southeast Asia. Comparative analysis of prM–E proteins identified 16 (2019) and 11 (2023) amino acid substitutions relative to the vaccine strain, and E-protein epitope mutation (N52D) is predicted to alter binding to the neutralizing antibody 1F4. These findings establish a molecular baseline for origin tracing and proactive surveillance in northwestern China.

Significance Dengue is increasingly reported in China, yet how the virus reaches inland regions with little local spread—and whether current vaccines and antibody treatments are well matched—remains unclear. By decoding and comparing dengue genomes from patients in Ningxia with global sequences, we show 2 separate arrivals in 2019 and 2023 likely seeded from Southeast Asia via southern travel hubs, and we identify changes in parts of the virus that antibodies target. These results trace likely entry routes to guide targeted traveler screening and mosquito control in low-transmission areas while highlighting genetic differences that should be considered when planning vaccination and antibody-based prevention or treatment.

## Introduction

Dengue fever, caused by dengue virus (DENV; genus *Flavivirus*, family *Flaviviridae*), is transmitted primarily by *Aedes aegypti* and *Aedes albopictus* (Pierson and Diamond 2020). Globally, an estimated 390 million infections occur annually, and approximately 3.9 billion people in 129 countries are at risk (Bhatt et al. 2013; Waggoner et al. 2016). The World Health Organization has reported a marked, approximately 10-fold increase in incidence over recent decades, with Asia bearing about 70% of the burden—particularly India, Indonesia, and the Philippines. In 2023, many regions experienced unprecedented surges characterized by rising incidence, geographic expansion, and emergence in previously unaffected areas (World Health Organization 2024).

China has experienced 3 outbreaks of dengue, with major epidemics in 1978 (22,122 cases, in Guangdong Province), 2014 (46,864 cases nationwide), and 2019 (15,171 cases, in a multi-province outbreak) (Wu et al. 2010; Lai et al. 2015). The 2014 outbreak, the largest to date, prompted nationwide public health reforms, including strengthened *Aedes* mosquito control measures, public awareness campaigns, real-time epidemic reporting systems, and interregional collaboration (Li et al. 2024: 2005–2023). While dengue cases were historically confined to southern China prior to 2000, rapid economic development, transportation infrastructure expansion, and increased tourism have facilitated its spread inland. Dengue is now reported in all Chinese provinces except Xizang (Tibet) (Wu et al. 2022), with the co-circulation of all 4 DENV serotypes documented and DENV-1 predominating since 2014 (Wu et al. 2010).

DENV infection ranges from asymptomatic or mild febrile illness to severe dengue (historically termed dengue hemorrhagic fever/dengue shock syndrome) (Halstead 1988). Diagnosis relies on NS1 antigen detection, IgM/IgG serology (ELISA), and RT-PCR (Peeling et al. 2010). Management remains supportive—particularly judicious fluid therapy—while prevention hinges on integrated vector management (source reduction, container control, and targeted larval/adult control) and community engagement (Simmons et al. 2012 Apr 12). Increasingly, genomic surveillance complements entomological and sero-epidemiological monitoring to resolve transmission dynamics.

The DENV genome is a single-stranded positive-sense RNA of approximately 10.7 kilobases in length. It contains a single open reading frame (ORF), flanked by 5′ and 3′ untranslated regions (UTRs) that play a crucial role in viral RNA replication and translation (Iglesias and Gamarnik 2011). The ORF encodes 3 structural (C, prM, E) and 7 nonstructural proteins (NS1, NS2A, NS2B, NS3, NS4A, NS4B, and NS5) (Pierson and Diamond 2020). The E protein mediates receptor binding and low-pH-triggered endosomal fusion and is the principal target of neutralizing antibodies (Bressanelli et al. 2004). Genotypic characterization using E-gene or whole-genome sequencing (WGS) underpins serotype/genotype assignment, phylogenetics, and outbreak investigation (Jing and Wang 2019). Open platforms (e.g. GISAID, GenBank) and phylodynamic tools (e.g. Nextstrain; BEAST—Bayesian evolutionary analysis by sampling trees) enable real-time reconstruction of transmission and source attribution (Hadfield et al. 2018).

Two dengue vaccines are currently licensed: CYD-TDV (Dengvaxia) is restricted to seropositive individuals due to the risk of antibody-dependent enhancement in seronegative recipients, whereas TAK-003 (Qdenga) has been approved in several jurisdictions since 2022 (Tricou et al. 2024). Potent human monoclonal antibodies such as HMAb 14c10 and 1F4, which target DENV-1 E-protein epitopes, illustrate mechanisms of neutralization at distinct stages of viral entry (Teoh et al. 2012; Fibriansah et al. 2014). Nevertheless, outbreak control in most settings still depends on timely surveillance and vector management.

The Ningxia Hui Autonomous Region is a historically low-risk, temperate region for dengue transmission. During nationwide upswings in 2019 and 2023, imported dengue cases were detected in Ningxia. To address the paucity of genomic data on introductions into low-risk regions, we performed WGS of 9 imported cases from these 2 upswings. We combined phylogenetics and phylodynamics (including genotype assignment and TMRCA/geographic origin inference) to characterize the viruses and contextualize them within regional and global circulation. Our findings provide critical insights into DENV transmission dynamics and genomic epidemiology, offering a framework to strengthen surveillance and outbreak preparedness in traditionally low-risk regions.

## Results

### Sample Background

Among the 9 samples analyzed, 5 (55.6%) tested positive for DENV-1 via RT-PCR. Three confirmed cases from 2023 were epidemiologically linked to travel to Xishuangbanna, Yunnan Province, China, with reported exposure to mosquito bites preceding symptom onset. Clinical manifestations in these individuals, occurring 7–9 d post-exposure, included fever, headache, fatigue, and periorbital edema. The remaining 2 positive cases, collected in 2019, lacked detailed epidemiological data (Table 1).

**Table 1:** Demographics and clinical backgrounds of patients with positive samples

Sample ID	Sex	Age	Travel history (location)	Travel dates	Clinical manifestations	Time from mosquito bite to onset of illness (day)	Body temperature (°C)	Severity classification	Collection Year	PCR results (C_T_)
23L3	Female	38	Xishuangbanna, Yunnan	2023-08-28	Fever, headache, fatigue, swollen eyes	7	38.5	Dengue without warning signs	2023	17
23L4	Male	31	Xishuangbanna, Yunnan	2023-09-27	Fever, headache, arthralgia	9	39.5	Dengue without warning signs	2023	18
23L5	Male	37	Xishuangbanna, Yunnan	2023-07-20	Fever, headache	7	39	Dengue without warning signs	2023	33
1902	−	−	−	…	−	−	−	−	2019	20
1903	−	−	−	…	−	−	−	−	2019	19

Dashes (−) indicate data was not collected.

### Whole-Genome Sequencing and Analysis

Of 5 DENV positive samples, 4 samples were successfully sequenced, thereby generating complete genomes. However, the remaining sample (23L5) failed to generate a complete genome (genome coverage 87.7%), which is likely due to low viral load. Sequencing on the Illumina MiSeq platform generated 2,605,748 to 4,666,740 paired-end reads per sample, with 80.5% to 97.64% of the reads mapped to the reference genome (DENV-1, NC_001477). Genome coverage for the 4 sequenced isolates ranged from 87.7% to 97.76%, yielding near-complete genomes with lengths of 9,415–10,502 base pairs (bp). All genomes retained a full-length ORF of 10,179 bp (except for 23L5), confirming intact coding regions (Table S3).

The amino acid divergence analysis identified 97 amino acid substitutions distributed across the ORFs. These substitutions occurred in both structural and non-structural proteins when compared to the DENV-1 standard strain (EU848545-Hawaii-1944). The segment exhibiting the highest proportion of substitutions was C-prM (4.6%), followed by E (3.4%), while NS5 exhibited the lowest (1.3%; Table 2).

**Table 2:** Amino acid substitutions in DENV ORFs

Structural proteins				Nonstructural proteins									
C-prM		E		NS1		NS2		NS3		NS4		NS5	
AA substitution	N	AA substitution	N	AA substitution	N	AA substitution	N	AA substitution	N	AA substitution	N	AA substitution	N
G9A	4	N8S	4	S94A	4	L41F	4	G44N	4	K39R	4	H127Y	2
S27P	2	K52D	4	T98A	4	A62G	2	K74R	2	R76K	2	N285H	4
M51I	4	T155S	4	R101K	4	H143Y	2	F85L	4	V97M	2	R325K	4
L72F	4	I161T	4	G105R	4	A148T	2	R255K	4	T177A	2	R378K	2
N75S	2	S171T	4	P181H	4	F159V	2	A293S	4	R184H	4	I392V	2
N90S	2	E203G	2	V224I	4	V168M	4	D350E	4	I259T	4	V413I	2
S93N	4	S225A	2	S261F	4	K218R	2	Q466H	4	T135I	2	K551R	2
T103A	2	P227S	4	D278G	2	I277V	4	G515E	4	T135M	2	T565E	2
L109M	4	E234Q	4	K324R	4	I330L	4	S517N	2	H200Y	2	T565K	2
I150L	4	K277T	4	D340A	2	K345R	2	K562R	4	…	…	S566A	2
R169Q	2	N290D	4	D340E	2	…	…	Q606L	4	…	…	V616A	4
K172E	4	V312L	2	L349M	4	…	…	…	…	…	…	G626R	2
A173T	2	V324I	4	…	…	…	…	…	…	…	…	E640K	2
R196Q	2	V380I	4	…	…	…	…	…	…	…	…	H649Y	4
A221T	4	L402F	4	…	…	…	…	…	…	…	…	T669I	4
K236R	4	I461V	4	…	…	…	…	…	…	…	…	I784V	4
I250M	2	A473T	4	…	…	…	…	…	…	…	…	V787I	2
…	…	M484L	4	…	…	…	…	…	…	…	…	V829I	4
…	…	…	…	…	…	…	…	…	…	…	…	E833G	2
…	…	…	…	…	…	…	…	…	…	…	…	L894P	4

AA, amino acid; N, number of samples; ORF, open reading frame.

### Phylogenetic Analysis

**Fig. 1.**
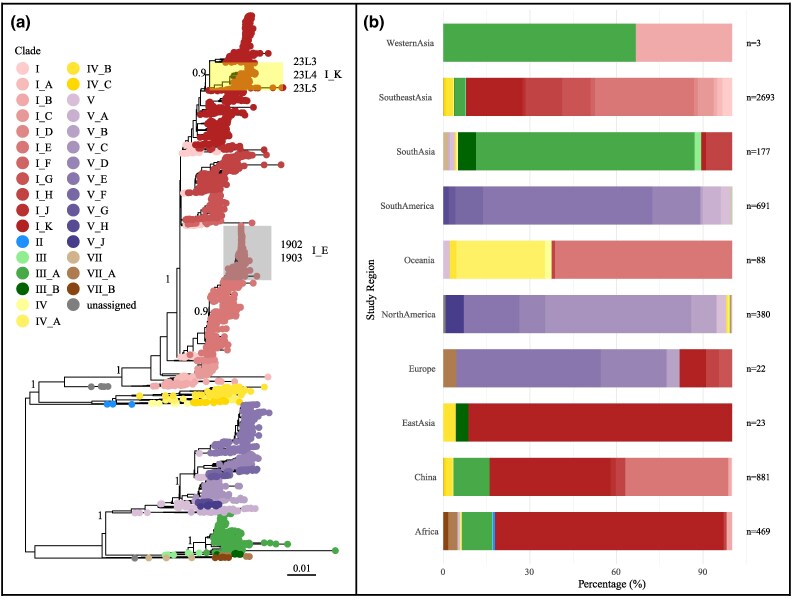
Maximum-likelihood phylogenetic tree of DENV-1 constructed using 4 Ningxia sequences, 876 publicly available sequences from China, and 3,642 sequences from other countries, all downloaded from GenBank. (a) Maximum-likelihood phylogenetic tree of the DENV-1 ORF constructed using IQ-TREE. Sequences from Ningxia in 2023 and 2019 are highlighted in yellow and gray, respectively. (b) Distribution of DENV-1 sequences by study region, with the number of sequences (*n*) indicated for each region.

**Fig. 2.**
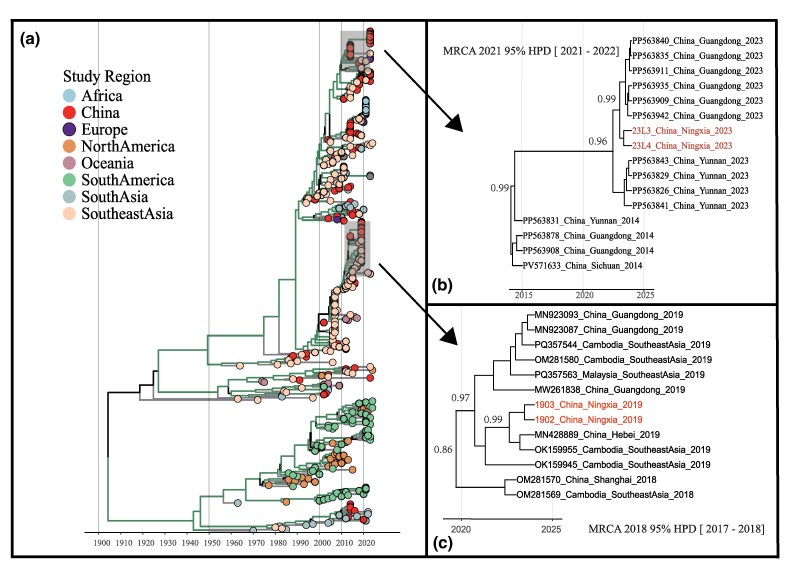
Bayesian time-scaled phylogenetic tree generated from phylogeographic analysis, incorporating 4 study sequences and publicly available sequences from GenBank. (a) Time-scaled phylogenetic tree of 452 DENV-1 ORF sequences constructed using BEAST v1.10.4, with tips colored by study region. Branches are colored by posterior probability support (green indicates posterior probability ≥0.8). (b, c) Enlarged views of the clades containing Ningxia sequences from 2023 and 2019, respectively. Posterior probability values are shown on key branches.

A maximum likelihood (ML) phylogenetic tree of DENV-1 was constructed using IQ-TREE, incorporating a comprehensive global dataset. The analysis assigned the 2019 and 2023 Ningxia strains to genotype I E (2019) and genotype I K (2023), respectively (Fig. 1a). The global distribution of DENV-1 genotypes exhibited marked regional differences, with distinct genotypes predominating in different geographic areas. For example, genotype I and its subclades are most prevalent in Southeast Asia, East Asia, Africa, and China, whereas genotypes V was mainly found in Europe, South and North America (Fig. 1b and Fig. 2a).

**Fig. 3.**
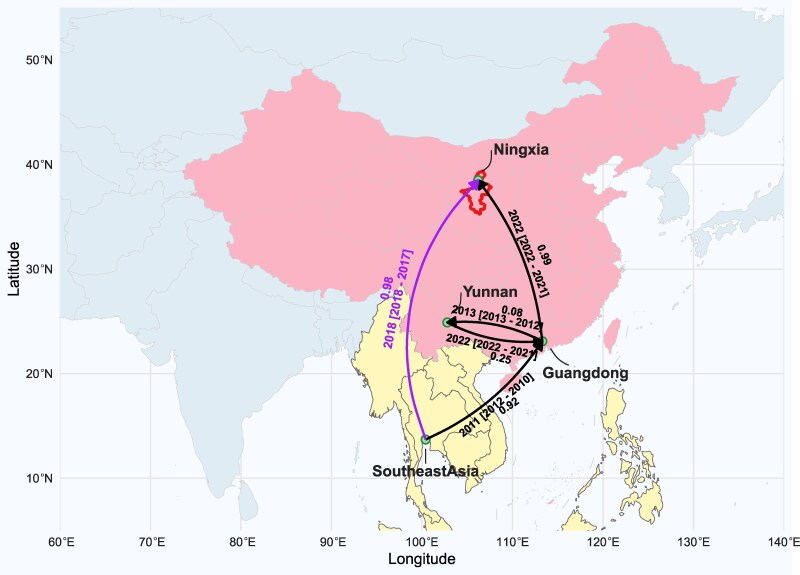
Spatiotemporal transmission map of DENV-1, illustrating inferred migration pathways and estimated years of transmission to Ningxia. The black line represents the 2023 Ningxia strain, while the purple line represents the 2019 Ningxia strain. The 2019 strain was likely introduced directly into Ningxia from Southeast Asia. In contrast, the ancestor of the 2023 strain was first introduced from Southeast Asia into Guangdong, subsequently circulated between Guangdong and Yunnan, and was ultimately transmitted to Ningxia. Each arrow represents a geographic transition event, corresponding to a node on the MCC tree where movement between 2 locations is inferred. The lines are annotated with the estimated date of the most recent common ancestor (MRCA) and the associated 95% high posterior density (HPD) intervals.

**Fig. 4.**
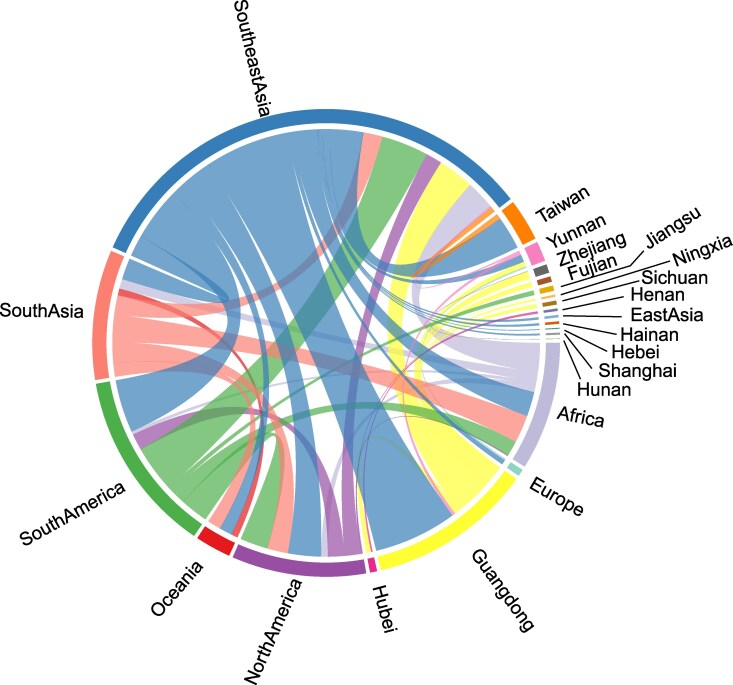
Markov jump history of DENV-1 migration events. Chord diagram based on Markov jump history, illustrating inferred DENV-1 migration events between study regions. The width of each link indicates the relative frequency of migration events between regions.

Bayesian phylogeographic analysis revealed that the 2019 Ningxia strain was closely related to a 2019 strain from Hebei Province, China (GenBank: MN428889), while the 2023 Ningxia strain clustered with strains from Yunnan and Guangdong provinces (Fig. 2b and c). The 2019 Ningxia strain and the 2023 Ningxia strain formed distinct monophyletic clades, with their most recent common ancestor (tMRCA) estimated to be in 1989 (95% highest posterior density [HPD]: 1985–1993). Spatiotemporal analysis demonstrated that the 2019 strains were likely introduced directly into Ningxia from Southeast Asia. In contrast, the ancestor of the 2023 strains was first introduced from Southeast Asia into Guangdong, subsequently circulated between Guangdong and Yunnan and was then inferred to be transmitted to Ningxia (Fig. 3). Markov jump analysis further revealed that Southeast Asia is the dominant source of DENV-1 migration events, with connections to multiple regions in China, including Guangdong, Yunnan, Hainan, Hubei, and Shanghai. Guangdong is the primary source of transmission to other provinces in mainland China, including Zhejiang, Fujian, Sichuan, Henan, and Ningxia. This highlights the central role of Southeast Asia in the transmission of DENV-1 to China (Fig. 4).

### Vaccine Strain Divergence and Antigen–Antibody Docking Analysis

**Fig. 5.**
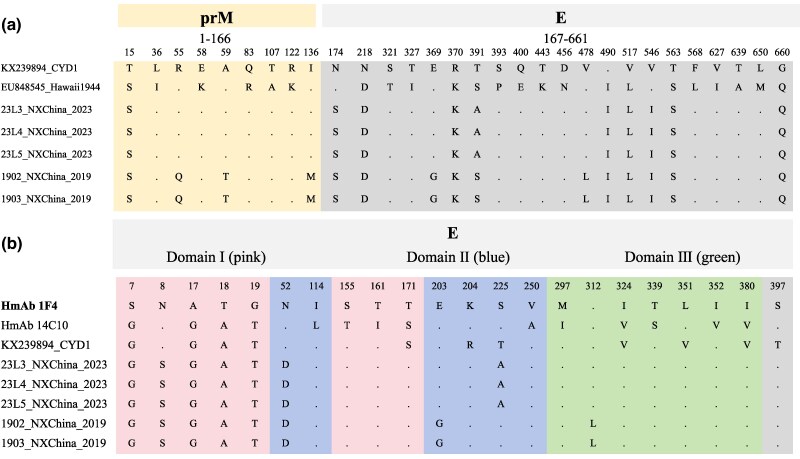
Comparative analysis of the amino acid sequences of the dengue virus serotypes 1. a) Alignment of the prM and E protein sequences of Dengvaxia (boldface) with the Hawaii strain and Ningxia. Only the amino acid positions that exhibit discrepancies are illustrated; prM and E protein sequences are shaded in red and gray, respectively. Numerals represent the protein amino acid position. b) Amino acid mismatch comparison between Ningxia dengue virus genomes and virus-neutralizing HmAbs. The amino acid changes presented are expected to disrupt binding between the envelope protein and heavy chain of the monoclonal antibodies (boldface). CYD, Dengvaxia vaccine; E, envelope; prM, premembrane; HmAb, human monoclonal antibodies.

Comparative analysis of the 2019 and 2023 DENV strains against the Dengvaxia® vaccine strain revealed pronounced amino acid divergence in the prM and E protein regions. The prM-E segments of the 2019 and 2023 strains displayed 16 and 11 amino acid substitutions, respectively, relative to the vaccine strains, corresponding to sequence divergences of 2.42% (2019) and 1.66% (2023). The 2019 strain displayed 4 substitutions in the prM region, whereas the 2023 strain exhibited only one (Fig. 5a).

Sequence alignment of the DENV E protein (co-crystallized with human monoclonal antibodies 1F4 and 14c10) with the Ningxia strain revealed mutations in the E protein. Compared with HMAb 14C10, 14, and 13 amino acid mismatches were identified in the 2019 and 2023 strains, respectively. In contrast, mismatches with HMAb 1F4 were fewer, limited to 8 residues (2019) and 7 residues (2023). Notably, all Ningxia strains shared an N52D mutation located within a known antigenic epitope, which may influence antibody recognition. Furthermore, key antigenic sites (E155, E161, and E171) in the Ningxia strains differed from those of HMAb 14C10 but were identical to those of HMAb 1F4 (Fig. 5b).

**Fig. 6.**
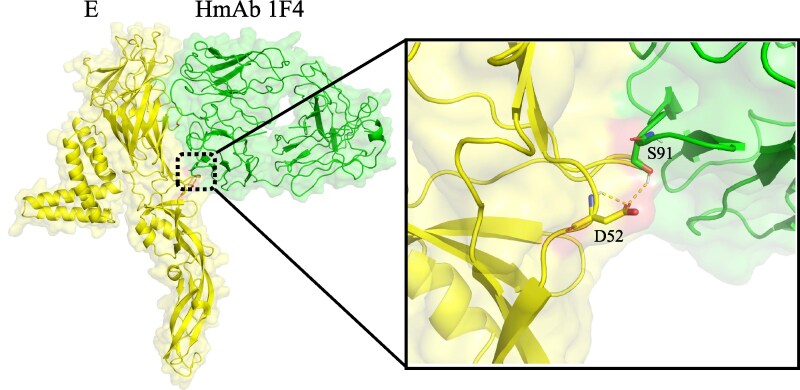
Molecular docking of the dengue virus envelope protein with neutralizing human monoclonal antibodies.

Docking analysis of the E protein from the Ningxia strains with the monoclonal antibody 1F4 revealed effective binding for all strains, supported by favorable binding energies (Table S6). Notably, the N52D mutation within the epitope altered the paratope interaction, shifting the key binding residues from S49 and G53 to S91 (Fig. 6). In contrast, docking with the antibody 14C10—which targets the E protein dimer—was not pursued, as the AlphaFold3-predicted dimer structure showed lower confidence in relevant interface regions (Table S5).

## Discussion

Imported dengue cases, driven by international travel and domestic migration, pose a growing public health challenge in China, particularly in regions with no established local transmission cycles (Fang et al. 2018: 2014–16). Between 2006 and 2020, China reported 81,652 indigenous and 12,701 imported dengue cases (Zhao et al. 2023). Genetic characterization of DENV strains in central and western China remains sparse, limiting insights into viral origin and transmission dynamics and underscoring the need for increased surveillance in low-endemic areas. We generated 5 near-full-length DENV-1 genomes from Ningxia, a non-traditional dengue transmission area in northern China, and integrated time-scaled phylogenetic and spatiotemporal diffusion models to infer 2 independent introduction events in 2019 and 2023. The Ningxia isolates from these 2 years fell within genotype I but belonged to distinct sublineages—I-E (2019) and I-K (2023). The 2023 Ningxia strains cluster closely with recent isolates from Guangdong and Yunnan. The 2019 Ningxia strains are grouped with sequences from Guangdong, Hebei, and Shanghai, as well as several from Southeast Asia (Cambodia and Malaysia). This is consistent with Sun's research findings that Myanmar and Cambodia were the main countries of origin for imported cases, and that Guangdong and Yunnan were important hubs for the domestic spread of DENV-1 within China (Ni et al. 2024).

In conjunction with patient travel history to Xishuangbanna, Yunnan, with documented mosquito exposure (Table 1), Bayesian phylogeographic reconstruction supports Southeast Asia as the upstream source of the Ningxia introductions: the 2019 event was possibly a direct importation, whereas the 2023 event likely reached Ningxia following domestic circulation in Guangdong. A multi-step route involving Yunnan was less well-supported by the data (Fig. 3). Markov-jump analyses further indicate that Southeast Asia constitutes the principal external source of introductions into mainland China, with Guangdong functioning as a hub within the interprovincial dissemination network—consistent with prior molecular epidemiological evidence on cross-border and interprovincial dengue spread in China (Cui et al. 2022; Cao et al. 2023). The time-calibrated phylogeny revealed that the Ningxia strain shares tMRCA with strains from Southeast Asian from around 1989, indicating that genotype I may have circulated cryptically in the southern China and Southeast Asian countries over the past 3 decades, evolving into various subgenotypes. Our findings are consistent with previous studies, demonstrating that dengue cases, often imported from Southeast Asian countries, are the primary source of local outbreaks in China (Cui et al. 2022: 1978–2017). For Ningxia, this transmission landscape underscores the need to prioritize surveillance for travel-associated importations from South–Southwest China and to account for compounded risks arising from climate warming and increasing transport connectivity that jointly shape vector ecology and human mobility.

Guntur Fibriansah found that Fab 1F4 molecules bind to domain I (DI), and DI-DII hinge of the E protein, with 26 amino acids of DENV1 E protein potentially interacting with Fab 1F4 (Fibriansah et al. 2014). We have identified a mutation in the Ningxia strain, N52D (Fig. 5b), which may affect Fab 1F4 neutralization, as N52 primarily matches S49 and G53 of the Fab 1F4 L chain. Our docking analysis further revealed that this N52D mutation alters the paratope interaction, shifting the key binding residues from S49 and G53 to S91 (Fig. 6). The precise effect of this altered interaction on the neutralizing capacity of antibody 1F4 requires future experimental validation. While many of these mutations may be linked to virulence, pathogenicity, and vector competence, the scope of this study did not encompass such functional investigations. K47E and G274E mutations have been reported to alter viral surface charge, potentially leading to antibody rejection and HMAb 1F4 neutralization escape (de Alwis et al. 2012). Mutations at these 2 loci were absent in Ningxia strains (Fig. 5b). The 2 monoclonal antibodies are likely effective in neutralizing the Ningxia strains presented here. However, further functional research is needed to clarify the impact of diverse genotypes and subtle amino acid variations in prM and E proteins on clinical immunity.

Ningxia, situated in northwestern China, encompasses diverse ecological landscapes, including the northern Yinchuan Plain within the middle reaches of the Yellow River. This region is characterized by an extensive network of lakes and wetlands, creating favorable ecological conditions for arthropod vector proliferation. While *Ae. aegypti*, the primary DENV vector, has not been documented in Ningxia to date, recent studies have identified the presence of *Aedes* (He et al. 2021; Fan et al. 2022; Yu et al. 2025). This finding raises important questions about the potential role of mosquito species in *flavivirus* maintenance or transmission within non-endemic regions. The absence of *Ae. aegypti* suggests that classical dengue fever transmission cycles are unlikely to establish in Ningxia under current conditions. However, the detection of *Aedes* highlights the need to investigate whether these mosquitoes could serve as secondary vectors for emerging *flaviviruses* or contribute to enzootic transmission cycles. Such ecological niches may facilitate viral adaptation or spillover, particularly in the context of climate change and increasing human encroachment into natural habitats.

The One Health framework, integrating human, animal, and environmental health, offers a multifaceted strategy to combat DENV transmission (Stadtländer 2015). Central to this approach is the establishment of integrated surveillance systems that synergize human syndromic monitoring, *Aedes* vector tracking, and environmental data (Procopio et al. 2024). In non-endemic regions like Ningxia, where imported cases pose risks, mobile health technologies, and AI-driven predictive tools can enhance outbreak forecasting by analyzing travel patterns, climate variables, and entomological indices. Such systems enable early detection and rapid response, critical for curbing secondary transmission. Future studies should prioritize longitudinal, multiyear surveillance incorporating asymptomatic cases, returning travelers, and local mosquito populations.

This study has several limitations. First, the small sample size (*n* = 9), collected across 2 non-consecutive years (2019 and 2023), limits the statistical power to comprehensively characterize the genetic diversity and transmission dynamics of DENV in Ningxia. Such sparse temporal sampling may not capture key evolutionary trends or emerging lineages. Second, reliance on hospital-based case reporting introduces surveillance bias, as asymptomatic or mild infections—which can contribute substantially to silent transmission—are systematically underrepresented. Third, the absence of entomological data (e.g. Aedes mosquito abundance, vector competence) precludes a robust assessment of local transmission risk and hinders validation of inferred spillover pathways. Fourth, although informative, the phylogenetic analyses depend on publicly available GenBank sequences, which may introduce biases in ancestral reconstruction and geographic attribution. Finally, the in silico antigen–antibody docking results should be interpreted with caution; the predicted impact of the identified mutations (e.g. N52D) on neutralizing efficacy requires experimental validation through neutralization assays. Moreover, the docking analysis was limited to the monomer-binding antibody 1F4, as the low-confidence interface prediction (ipTM score) for the E-protein dimer precluded reliable docking with the dimer-binding antibody 14C10.

## Conclusions

This study presents the first genomic characterization of autochthonous DENV type 1 (DENV-1) from northwestern China. Phylogenetic analysis suggests independent introduction events, likely directly from Southeast Asia in 2019 and through domestic circulation via Guangdong in 2023. The Ningxia strains exhibit antigenic divergence from the vaccine strain, and an E-protein epitope mutation (N52D) is predicted to alter binding to the neutralizing antibody 1F4. This provides an important molecular basis for the origin tracing, transmission surveillance, and control strategies of the DENV in northwestern China.

## Materials and Methods

### Surveillance and Sample Collection

Ningxia Hui Autonomous Region is a non-endemic area for dengue fever, with all confirmed cases classified as imported. Case detection primarily occurs through hospital-based surveillance. When a patient presents to a healthcare facility, clinicians conduct a preliminary diagnosis based on clinical symptoms. Suspected dengue cases are reported to the local Centers for Disease Control and Prevention (CDC) in accordance with the National Dengue Surveillance Program. Subsequently, CDC personnel perform an epidemiological investigation and coordinate with the hospital to collect the patient's blood samples. These samples are then transferred to the Ningxia Hui Autonomous Region CDC laboratory for confirmatory testing, including DENV nucleic acid detection and NS1 antigen assays.

### Study Samples and Processing

The human serum samples analyzed in this study were derived from dengue surveillance in Ningxia during 2019 and 2023. Over these 2 years, our laboratory received 9 blood samples from hospital-reported suspected dengue cases. Five of the samples tested positive for NS1 protein as well as nucleic acids. All samples were stored at −80 °C prior to next-generation sequencing (NGS) analysis.

### Detection of Dengue Virus

Viral RNA was extracted from 200 μL serum samples using the High Pure Viral RNA Kit (Roche; catalog no. 11858882001) according to the manufacturer's protocol. Briefly, serum samples were lysed by incubation with lysis buffer for 10 min at room temperature, with intermittent mixing to ensure complete homogenization. The lysate was then passed through a silica membrane column for RNA binding. After 2 wash steps to remove contaminants, purified RNA was eluted in 50 μL of nuclease-free water. A real-time RT-PCR method with TaqMan probes was employed to estimate the concentration of target templates and confirm the DENV serotype of the RNA samples. Reactions were carried out using the HiScript II One Step qRT-PCR Probe Kit (Vazyme, catalog no. Q222-01) with DENV-specific primers and probes (Table S1), as per the protocol recommended by the National Health Commission of the People's Republic of China (National Health and Family Planning Commission 2018). Each run included a positive control (confirmed DENV RNA-positive clinical samples) and a negative control (nuclease-free water) to validate assay specificity and sensitivity. Thermal cycling conditions were as follows: reverse transcription at 50 °C for 15 min, initial denaturation at 94 °C for 30 s, followed by 40 cycles of 95 °C for 10 s and 60 °C for 30 s. Fluorescence signals were acquired during the annealing/extension phase.

### Whole Genome Sequence

The complete DENV genome was sequenced using a NGS approach adapted from Wen et al. (Su et al. 2022). Briefly, cDNA was synthesized from purified viral RNA using the LunaScript® RT SuperMix Kit (New England BioLabs, catalog no. E3010). The entire viral genome was amplified via multiplex PCR with DENV-specific primers (Table S2) and Q5 Hot Start High-Fidelity DNA Polymerase (New England BioLabs, catalog no. M0493L) to ensure amplification accuracy. PCR products were purified using AMPure XP beads (Beckman Coulter, catalog no. A63881) according to the manufacturer's protocol. Purified DNA was quantified with a Qubit 4.0 fluorometer (Invitrogen) and the Qubit dsDNA High Sensitivity (HS) Assay Kit (Invitrogen). Sequencing libraries were constructed using the Nextera XT DNA Library Preparation Kit (Illumina) following the manufacturer's guidelines. Libraries were normalized, pooled, and sequenced on an Illumina MiSeq platform with a 300-cycle reagent cartridge, generating paired-end reads (2 × 150 bp) for downstream analysis.

### Data Analysis

Raw sequencing data (Fastq files) were processed using CLC Genomics Workbench 24 (CLC bio/Qiagen, Aarhus, Denmark), a platform optimized for viral genome assembly and variant detection in low-input clinical samples. Quality control included trimming reads shorter than 70 nucleotides (nt) and removing adapter sequences from both ends. Primer sequences were subsequently trimmed to exclude amplification artifacts. The filtered reads from each sample were then mapped to the DENV reference genome (NC_001477 DENV-1) utilizing the software's recommended workflow and default parameters. Finally, the genome coverage, sequencing depth, and consistency of the consensus sequences were evaluated to ensure robust analytical outcomes.

### Time-scaled Phylogenetic Tree Construction Using BEAST

A total of 5,422 DENV-1 whole-genome sequences from China and global regions were retrieved from the National Center for Biotechnology Information (NCBI) database. Four DENV-1 whole genomes newly sequenced from Ningxia were incorporated into the dataset, along with additional similar sequences identified using the NCBI BLASTN tool. All sequences were aligned using MAFFT (v7.520), and the 5′ and 3′ UTRs were trimmed to retain only the ORF. To construct a phylogenetically representative dataset, stratified proportional sampling was performed based on genetic distances between the 4 Ningxia sequences and global regions, resulting in a final dataset of 452 sequences. The dataset comprised 22 distinct geographic units, including 14 provinces from China and 8 international regions, with viral sequences collected between 1944 and 2024. Phylogenetic reconstruction was conducted using IQ-TREE (v2.3.5) with the best-fit substitution model (GTR + F + I + R4), as determined by ModelFinder. ML trees were inferred with 1,000 ultrafast bootstrap replicates, and branch support values were calculated accordingly.

The phylogeographic dynamics of DENV-1 were inferred using BEAST (v1.10.4), which simultaneously estimated phylogenetic tree topology and migration patterns by implementing the Bayesian Stochastic Search Variable Selection approach. Ancestral state reconstruction was also performed. The final dataset was analyzed using a general time-reversible (GTR) nucleotide substitution model with a gamma-distributed prior for each relative substitution rate. A relaxed uncorrelated lognormal molecular clock model, with a gamma-distributed prior for the average clock rate, was used to infer the timescale of DENV-1 evolution. The maximum clade credibility (MCC) tree was annotated using TreeAnnotator (included in the BEAST package). Sequence from the current study were genotyped using the Nextclade v3.16.0 (https://clades.nextstrain.org), and genotypes were displayed on the phylogenetic tree. Markov jump counts were recorded throughout the phylogenetic history to highlight migration events relevant to the dispersal and evolution of lineages within China and global regions. All supplementary analyses and visualizations were conducted in R (version 4.1.2). The complete set of XML files generated during these analyses, along with the R code for plotting, is publicly accessible at https://github.com/bmwlongshan/Dengue.

### Structural Modeling and Antigen-antibody Docking Analysis

The amino acid sequences of the DENV envelope (E) protein in complex with the human monoclonal antibodies (mAbs) 1F4 (PDB ID: 4C2I) and 14c10 (PDB ID: 4CAU) were obtained from the Protein Data Bank. These reference sequences were aligned with the translated E protein sequences from the 5 Ningxia isolates obtained in this study. To assess sequence conservation within the antigenic sites, multiple sequence alignment of the E protein was performed using BioEdit.

The 3-dimensional structure of 5 Ningxia DENV-1 E proteins was predicted using AlphaFold 3 (https://alphafoldserver.com). The structures of the Fab fragments for mAbs 1F4 and 14c10 were extracted from their respective PDB entries. Protein–protein docking was performed using HADDOCK2.4, guided by the known epitope–paratope interactions defined in the crystallographic complexes. Active residues for docking were defined based on the crystal structure contacts, and passive residues were automatically defined by the software. The top-ranking cluster of solutions based on HADDOCK score was selected for subsequent analysis, and structural visualizations were prepared with PyMOL.

## Ethical Approval

This study was granted ethical approval by the Ethics Review Committee of the Ningxia Center for Disease Control and Prevention (Approval No. 2024-LLSC-104). Informed consent was waived due to the retrospective design and anonymized nature of the epidemiological data. All datasets were rigorously anonymized prior to analysis to protect participant confidentiality. Experimental procedures strictly adhered to China's national ethical guidelines and regulatory requirements for biomedical research.

## Supplementary Material

evag045_Supplementary_Data

## Data Availability

The nucleotide sequences of the Ningxia strains examined in this study were deposited in the GenBank database under the accession numbers PQ580142-PQ580145.
