# Supplementary material for: Genomic Characterization of Dengue Virus in the Ningxia Hui Autonomous Region, China (2019 and 2023)
Source: Genome Biol Evol. 2026 Feb 28;18(3):evag045. doi: 10.1093/gbe/evag045 (PMC12980784; doi:10.1093/gbe/evag045)
Supplement: evag045_Supplementary_Data [file evag045_supplementary_data.pdf]

TITLE

Genomic characterization of dengue virus in the Ningxia Hui Autonomous Region, China（2019 and 2023）

SUPPLEMNTARY METHODS

Recombination Detection using RDP4

Recombination, defined as the transfer of nucleic acid material between genomes, is a major driver of genetic diversity, alongside mutation and reassortment, providing the raw material upon which natural selection acts(1). The occurrence of recombination can result in different genomic regions exhibiting distinct evolutionary histories, thereby precluding the accurate representation of the entire genome's evolutionary trajectory by a single, bifurcating phylogenetic tree. This presents significant challenges for the inference of key evolutionary parameters in pathogens, such as molecular clock rates, selection pressures, and effective population sizes. Consequently, most phylogenetic analyses assume the absence of recombination within the sequences under study(2).

To address this, we used RDP4 (v.4.101) to screen for recombinant sequences(3). The initial sequence alignment was analyzed using seven primary recombination detection methods: RDP, BOOTSCAN/RESCAN, GENECOV, MAXCHI, CHIMAERA, SISCAN, and 3SEQ. A sequence was classified as recombinant and excluded from downstream analyses if at least five out of the seven methods identified it as a potential recombinant. As a result, 17 sequences were identified as putative recombinants and removed from further analysis: FJ196846, JQ922546, JQ922547, OR518260, FJ196843, FJ196847, FJ196848, JF459993, JN903578, KF289073, KU509254, MW582813, OM281589, ON109597, ON123666, PP563968, and PV554923.

Assessment and Optimization of DENV- 1 Phylogeny Using TempEst

The maximum likelihood (ML) phylogenetic tree generated by IQ-TREE was imported into the Tree panel of TempEst for visualization. Both the Root-to-tip and Residuals panels were utilized to identify sequences whose sampling dates were incongruent with their genetic divergence. These outlier points were manually selected, resulting in the corresponding sequences being highlighted across all panels in TempEst. By returning to the Tree panel, we were able to examine the labels and phylogenetic positions of these problematic sequences(4). In our dataset, four such sequences were identified—accession numbers: MT076934, MT076935, KU094071, and OR389282. Of these, three sequences fell below the regression line and one

above it, indicating that their genetic divergence was either smaller or larger than expected based on their sampling dates. Such discrepancies may arise from various sources, including errors in sequence assembly or misalignments in portions of the sequence, potentially resulting in anomalously long branches in the phylogeny. Regardless of the underlying cause, it is standard practice to exclude these sequences from the multiple sequence alignment prior to subsequent analyses (Figure S1).

Following the removal of these four outlier sequences, a new ML tree was reconstructed and re-imported into TempEst. In the Root-to-tip panel, a regression analysis was performed to assess the relationship between genetic divergence from the root and sampling time, with each point representing a time-stamped sequence. The Best-fitting root function was employed to select the root that minimized the sum of squared residuals. The correlation coefficient (R^2) provided an objective, albeit informal, measure of the temporal signal. Our analysis revealed a strong correlation ($R^2 = 0.89$) between genetic distance and sampling date in the phylogeny of dengue virus serotype 1, indicating that the dataset is well-suited for molecular clock analysis in BEAST. The slope of the regression line yielded an estimated evolutionary rate of 7.06×10^{-4} substitutions per site per year, while the intercept with the time axis suggested a root age of approximately 1915 (Figure S2). These estimates are consistent with previous reports for dengue virus, further validating our analytical approach(5, 6).

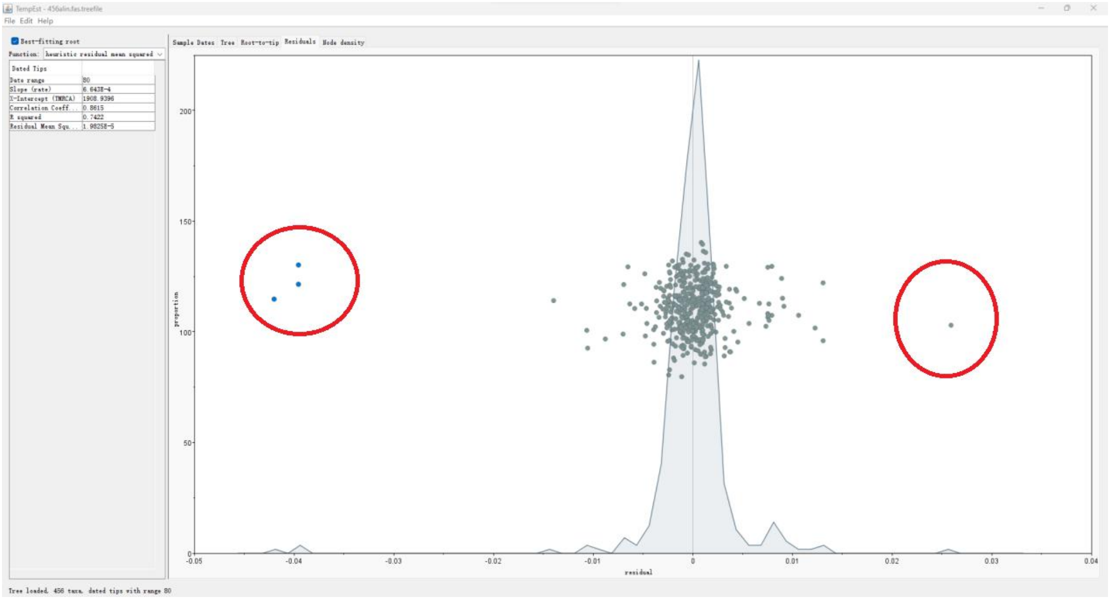

Figure S1: Residuals plot in TempEst. Four outliers can be identified and are highlighted with red
circles.

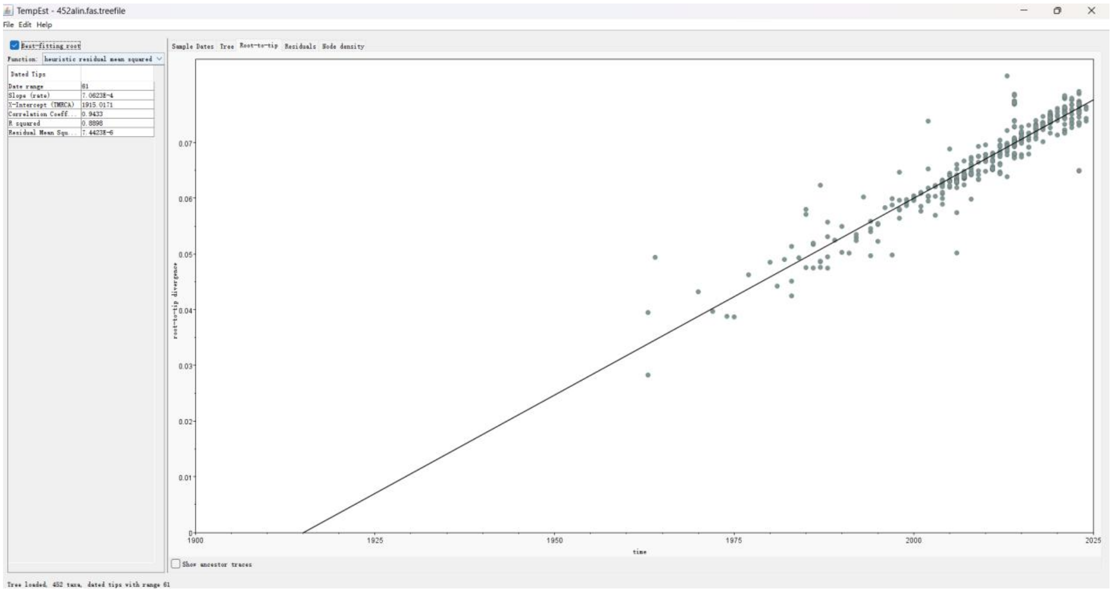

Figure S2. The temporal signal of the maximum likelihood phylogenetic tree constructed from 452 sequences was evaluated using TempEst. Collection years were assigned based on GenBank annotations. Sequences exhibiting significantly greater genetic divergence than expected were excluded from the analysis. Temporal regression analysis estimated the time to the most recent common ancestor (tMRCA) of the dataset to be 1915. The analysis yielded an R^2 value of 0.89, a correlation coefficient of 0.94, and a regression slope of 7.06×10^{-4} substitutions per site per year.

Table S1. The DENV-specific primers and probes recommended by the National Health Commission of the People's Republic of China used for real-time PCR in this study.

Name	Sequence (5'→3')	Fluorescent	Target
Den-FP	GCATATTGACGCTGGGAGAGA		
Den-RP	GGCGTTCTGTGCCTGGAAT		DENV 1-4
Den-PP	CAGAGATCCTGCTGTCTC	FAM/MGB	
Den-1F	CAA AAG GAA GTC GTG CAA TA		
Den-1C	CTG AGT GAA TTC TCT CTA CTG AAC C		DENV 1
Den-1P	CAT GTG GTT GGG AGC ACG C	FAM/BHQ-1	
Den-2F	CAG GTT ATG GCA CTG TCA CGA T		
Den-2C	CCA TCT GCA GCA ACA CCA TCT C		DENV 2
Den-2P	CTC TCC GAG AAC AGG CCT CGA CTT CAA	HEX/BHQ-1	
Den-3F	GGA CTG GAC ACA CGC ACT CA		
Den-3C	CAT GTC TCT ACC TTC TCG ACT TGT CT		DENV 3
Den-3P	ACC TGG ATG TCG GCT GAA GGA GCT TG	TesasRed/BHQ-2	
Den-4F	TTG TCC TAA TGA TGC TGG TCG		
Den-4C	TCC ACC TGA GAC TCC TTC CA		DENV 4
Den-4P	TTC CTA CTC CTA CGC ATC GCA TTC CG	Cy5/BHQ-3	

Table S2. The primers used for the whole genome sequencing of DENV-1 in this study. The final concentration of each primer was 0.015 μM.

Name	Direction	Sequence (5’→3’)	Position
DENV-1_1F	Forward	GTCTACGTGGACCGACAAGAAC	9-30
DENV-1_1R	Reverse	AGATCTCTTTCTTGAAACCYCGT	337-359
DENV-1_2F	Forward	CAGTTGGCGAAGAGATTCTCA	176-196
DENV-1_2R	Reverse	ACATAACTCTCCCAAATCCATYG	549-571
DENV-1_3F	Forward	GGAGAGCCACACATGATAGT	458-477
DENV-1_3R	Reverse	CCTATTCCCACGCATCGCAT	935-954
DENV-1_4F	Forward	TGATAGCCTTTTTTTTAGCACATGC	840-864
DENV-1_4R	Reverse	GTTTTCATATTGAACTATCTTTCCTTC	1310-1336
DENV-1_5F	Forward	ACGAACGTTTGTGGAYAGAGG	1213-1233
DENV-1_5R	Reverse	TTCTTGTGATCCCARTACRACYA	1683-1705
DENV-1_6F	Forward	TTTGCTAGTCACATTYAAGACAG	1639-1661
DENV-1_6R	Reverse	TGCTTCCTTTCTTRAACCAGCT	2102-2123
DENV-1_7F	Forward	TACTGACAAAGAAAAACCAGTCAAYATT	2008-2035
DENV-1_7R	Reverse	ATTAGTGACAAAAATGCCRCTTCCA	2464-2488
DENV-1_8F	Forward	CTRGTMACACTRTACCTAGGAG	2384-2405
DENV-1_8R	Reverse	TTTCAGCCATATGTTTGTYGTGA	2907-2929
DENV-1_9F	Forward	TTGAGACGACTACCTTCATYATYGA	2802-2826
DENV-1_9R	Reverse	GAGATGGTCCTCGATTTCCACA	3290-3311
DENV-1_10F	Forward	ACAGACCAGGATATTTCACACAAAC	3186-3210
DENV-1_10R	Reverse	CAACAGCAAACATYGGTCTCAT	3722-3743
DENV-1_11F	Forward	TATAATGGGACAAYTGACRTGGA	3604-3626
DENV-1_11R	Reverse	AACATGGTTAGTGGTTTRCATCC	4073-4095
DENV-1_12F	Forward	ATCATGATGTTAAAATTGTTGACYGA	3866-3891
DENV-1_12R	Reverse	GACCTCCACTAAGATRTTGTGT	4342-4363
DENV-1_13F	Forward	TCCTGGGAAGAAGAAGCAGA	4307-4326
DENV-1_13R	Reverse	CTCCAACCTCCTCCATATGAGA	4749-4770
DENV-1_14F	Forward	AGGGGAGCTGTCCTYATGTA	4679-4698
DENV-1_14R	Reverse	CTTTTTATGGCCTCACGRACTAT	5141-5163
DENV-1_15F	Forward	ATTGAGGACGARGTGTTYARGA	5054-5075
DENV-1_15R	Reverse	TCAGGAATGTCTCTTTCCTCATCTT	5520-5544
DENV-1_16F	Forward	AATCTTTATGACRGCYACYCC	5455-5475
DENV-1_16R	Reverse	TCGTTGTTTAAAGGCTGTCCCA	5946-5967
DENV-1_17F	Forward	GCAGAGGAAGAATTGGAAGGAAC	5892-5914
DENV-1_17R	Reverse	TAGATCACCTGAGACRCTTCT	6374-6394
DENV-1_18F	Forward	GAAGGAGAAAGAAAGAAACTRCGACC	6275-6300
DENV-1_18R	Reverse	CATGAATAACAAACCTATCACCAC	6785-6808
DENV-1_19F	Forward	AGTGTGGARCCCCATTGGAT	6671-6690
DENV-1_19R	Reverse	TTCTCTAGTACCTTTTGCTTGCA	7203-7225
DENV-1_20F	Forward	GGATGGCCAATATCRAARATGGA	7070-7092
DENV-1_20R	Reverse	CTACCTCCTCCTAAAGATTTCAT	7547-7569
DENV-1_21F	Forward	GGAAAATTCTGGAACACYACGAT	7457-7479

DENV-1_21R	Reverse	GCCATTGGATTAGGTTCYTCRT	7902-7923
DENV-1_22F	Forward	GAAGAGGTGGCTGGTCATAY	7821-7840
DENV-1_22R	Reverse	GTCCACGTCTCTTTCATAYGTTGG	8318-8341
DENV-1_23F	Forward	TCAGCAGTAAATATGACATCYAGAAT	8258-8283
DENV-1_23R	Reverse	ACTTGGCTGTTACCTCCATRAT	8687-8708
DENV-1_24F	Forward	ACAGAGGGTGTTTAAAGAGAAAGT	8623-8646
DENV-1_24R	Reverse	TCCAAGTTTGTGGAGTCCYTCTC	9096-9118
DENV-1_25F	Forward	GGAAGTCGYGCAATATGGTACAT	8978-9000
DENV-1_25R	Reverse	CAATTCGCTRGGTGAAAAGATTC	9450-9472
DENV-1_26F	Forward	TCATATCCAGACGTGACCAGA	9345-9365
DENV-1_26R	Reverse	TGGAAATACATCAGCTGCCACAT	9851-9873
DENV-1_27F	Forward	ATGCCGCAAYCAAGATGAACT	9751-9771
DENV-1_27R	Reverse	TCACTCTCGTTYTTGAATCTYTTCA	10227-10251
DENV-1_28F	Forward	CCACCAACATACAAGTRGCYATA	10149-10171
DENV-1_28R	Reverse	CTCCACTAACCACTAGTCTGCTA	10503-10525
DENV-1_29F	Forward	TCCAAGGACGTWAAATGAAGTCAG	10379-10402
DENV-1_29R	Reverse	GATTCAACRGCAYCATTCCAT	10705-10725

Table S3. The results of the second-generation sequencing, including the number of sequencing reads, the sequencing depth, and the concordant sequence coverage.

Sample ID	Serotype	Genotype	Total No.of reads	No. of passing QC	No. (%) of mapped reads	Average depth of coverage	Consensus length (bp)	Genome coverage %
23L3	DENV-1	Genotype I K	2605748	2602388	2501733 (96.13)	26442.57	10501	97.82
23L4	DENV-1	Genotype I K	2697616	2694736	2607167 (96.75)	36445.53	10502	97.83
23L5	DENV-1	Genotype I K	2805244	2803322	2258104 (80.5)	1326.2	9415	87.7%
1902	DENV-1	Genotype I E	4666740	2664520	2601532 (97.64)	52140.73	10400	96.88
1903	DENV-1	Genotype I E	4219014	4218568	4081798 (96.76)	48454.59	10495	97.76

Table S4. Statistical analysis of the nucleotides in the coding region of the Ningxia strain, including the percentage of each base and the G+C ratio.

Sample ID	A (%)	T (%)	C (%)	G (%)	Length (bp)	C+G content (%)
Hawaii1944	31.71	21.66	20.57	26.05	10179	46.63
23L3	31.91	21.51	20.62	25.97	10179	46.59
23L4	31.95	21.51	20.60	25.94	10179	46.54
1902	32.05	21.59	20.58	25.78	10179	46.36
1903	32.07	21.63	20.54	25.76	10179	46.30

Table S5. Prediction scores for E protein monomers of five dengue virus sequences by AlphaFold 3.

Sample ID	pTM	ipTM
23L3monomer	0.73	-
23L4monomer	0.73	-
23L5monomer	0.74	-
1902monomer	0.73	-
1903monomer	0.73	-
23L3dimer	0.61	0.57
23L4dimer	0.6	0.58
23L5dimer	0.6	0.58
1902dimer	0.6	0.57
1903dimer	0.6	0.57

pTM: Predicted template modeling score; ipTM: interface predicted template modeling score.

Table S6. Docking scores for antibody-antigen complexes predicted by HADDOCK2.4

	1f4-1903	1f4-1902	1f4-23L4	1f4-23L5	1f4-23L3
HADDOCK score	-124.6 (2.4)	-109.5 (2.5)	-123.0 (4.4)	-121.0 (6.3)	-133.8 (4.8)
Cluster size	57	22	26	26	36
RMSD from the overall lowest-energy structure	0.6 (0.4)	8.3 (0.4)	1.3 (0.8)	1.2 (0.8)	1.0 (0.7)
Van der Waals energy	-64.7 (8.4)	-64.1 (5.2)	-63.9 (3.7)	-63.3 (11.1)	-68.9 (6.5)
Electrostatic energy	-350.7 (36.8)	-285.1 (17.0)	-364.3 (14.4)	-376.4 (63.3)	-368.3 (46.6)
Desolvation energy	2.2 (4.3)	2.3 (0.5)	5.5 (3.2)	4.2 (7.2)	-2.1 (3.2)
Restraints violation energy	79.7 (19.4)	93.2 (30.0)	82.9 (24.2)	134.2 (18.1)	108.3 (22.0)
Buried Surface Area	2534.5 (53.5	2417.7 (111.9)	2463.8 (167.8)	2586.3 (209.6)	2658.9 (102.4)
Z-Score	-1.7	-1.4	-2	-1.8	-2.2

Note: Standard deviations are shown in parentheses.

SUPPLEMENTARY REFERENCES

1. Awadalla P. 2003. The evolutionary genomics of pathogen recombination. Nat Rev Genet 4:50–60.

2. Arenas M, Posada D. 2010. The effect of recombination on the reconstruction of ancestral sequences. Genetics 184:1133–1139.

3. Martin DP, Murrell B, Golden M, Khoosal A, Muhire B. 2015. RDP4: Detection and analysis of recombination patterns in virus genomes. Virus Evol 1:vev003.

4. Hill V, Baele G. 2019. Bayesian Estimation of Past Population Dynamics in BEAST 1.10 Using the Skygrid Coalescent Model. Mol Biol Evol 36:2620–2628.

5. Pyke AT, Moore PR, Taylor CT, Hall-Mendelin S, Cameron JN, Hewitson GR, Pukallus DS, Huang B, Warrilow D, Van Den Hurk AF. 2016. Highly divergent dengue virus type 1 genotype sets a new distance record. Sci Rep 6:22356.

6. Sun B, Xu M, Jia L, Liu H, Li A, Hui L, Wang Z, Liu D, Yan Y. 2025. Genomic variants and molecular epidemiological characteristics of dengue virus in China revealed by genome-wide analysis. Virus Evol 11:veaf013.
